# Barriers and facilitators to using self-sampled tests for the human papillomavirus (HPV): a mixed-methods study to inform a horizon scan

**DOI:** 10.1017/S0266462326103729

**Published:** 2026-04-13

**Authors:** Soheila Ghasri, Eugenie Evelynne Johnson, Bethan Molly Harris, Imogen Forsythe, Claire Eastaugh, Adetutu Sadiq, Nick Meader, Matthew Breckons, Fiona Pearson

**Affiliations:** NIHR Innovation Observatory, Newcastle University, UK; Population Health Sciences Institute, Newcastle University, UK

**Keywords:** methods, qualitative research, human papillomavirus, self-sampling, screening

## Abstract

**Objectives:**

Human papillomavirus (HPV), particularly high-risk types such as HPV 16 and 18, is a major cause of cervical cancer and other cancers. Despite the United Kingdom’s (UK’s) commitment to cervical cancer elimination by 2040, participation in HPV screening is declining, disproportionately affecting underserved groups, including those experiencing poverty, people from minoritized racial, ethnic, gender, or sexual identity groups, and people living with HIV.

**Methods:**

We conducted a mixed-methods study to explore awareness, barriers, and facilitators to HPV self-sampling from clinician and public perspectives. A multi-stakeholder survey (*n* = 105) and two online focus groups with clinicians (*n* = 4) and members of the public (*n* = 5) were undertaken.

**Results:**

Survey respondents identified accuracy, cost-free availability, ease of use, accessibility, clear instructions, and adequate follow-up as critical test features. Participants emphasized that disability, cultural context, language, and socioeconomic status strongly influence barriers and facilitators to uptake. Focus groups provided contextual depth, illustrating how privacy, logistical and emotional impacts, and supportive follow-up pathways shaped acceptability and trust. Clinicians highlighted the need for integration into healthcare infrastructure to maintain trust and ensure support. Public participants recommended community-driven engagement, including multilingual instructions and tailored communication to encourage adoption among diverse groups. Concerns were raised about unintended consequences, such as anxiety following asymptomatic HPV diagnoses and challenges in managing clinical pathways after positive results. Suggestions included leveraging community organizations to reduce hesitancy.

**Conclusions:**

Findings highlight policy and implementation considerations for embedding HPV self-sampling within care pathways to improve uptake and reduce inequalities.

## Introduction

The human papillomavirus (HPV) is a common sexually transmitted infection (STI) that often causes no symptoms and can affect the skin, genital area, and throat (1). There are more than 100 types of HPV; high-risk HPV types can be linked to cervical cancer, anal cancer, penile cancer, vulval cancer, vaginal cancer, and some forms of head and neck cancer (1). Most cervical cancer cases in the United Kingdom (UK) are caused by HPV (99.7 percent) (2). Testing for the virus is currently part of cervical screening, which is offered to all women and people with a cervix between the ages of 25 and 64 (1).

In England, the National Health Service (NHS) has pledged to eliminate cervical cancer by 2040 by improving access to HPV vaccinations and increasing screening uptake (3). However, it has been noted that screening uptake for women aged between 25 and 49 years old has decreased from 73.8 percent in 2009–10 to 67.5 percent in 2023–24, with a decrease from 78.7 percent to 74.9 percent for women aged 50 to 64 in the same period (4). Although some of the recent decreases in screening have been attributed to the effects of the COVID-19 pandemic (5), it has been noted that HPV screening uptake varies between groups. A longitudinal analysis of English general practices located in areas of higher deprivation reported lower screening rates compared to the least deprived quintile (6), whereas transgender men and nonbinary people assigned female at birth have been noted to experience barriers in accessing screening services (7).

Cervical screening is currently undertaken by clinicians, who use a speculum and brush to obtain a sample of cells from the cervix (8). However, systematic reviews have suggested that self-sampling for HPV may be an acceptable approach (9;10). Self-sampling is currently applied in the UK for screening for health conditions such as bowel cancer (11). Furthermore, previous studies have suggested that testing for HPV outside of a clinical setting may be convenient, removes embarrassment, is less invasive, and can enhance privacy (12;13).

As the precursor to a horizon scan to identify emerging self-sampled diagnostic tests for HPV (14), we aimed to explore the perspectives of both individuals and healthcare professionals to understand potential barriers and facilitators to their adoption, and to discuss possible health inequalities associated with self-sampled HPV testing. Horizon scanning uses a structured approach similar to evidence synthesis to identify emerging technologies (15); this work was intended to both explore these perspectives and guide the analysis and reporting of the subsequent horizon scan.

Through a stakeholder survey and two focus groups, this study aimed to address the following questions.

- What are the perspectives of the public and clinicians on self-sampled HPV diagnostic tests?
- How might HPV self-sampling unintentionally reinforce or reduce existing health inequalities, and what barriers and facilitators influence this?

## Methods

A mixed-method approach was undertaken, including a stakeholder survey followed by two focus groups with clinicians and members of the public. A completed Good Reporting of a Mixed Methods Study (GRAMMS) checklist is presented in Supplementary Material 1 (16).

### Ethical approval

Ethical approval for this study was obtained from Newcastle University on 13 August 2024 (reference: 2790/48097).

### Survey methods

#### Design

The survey was designed using the secure online survey platform Qualtrics; the final version can be found in Supplementary Material 2. The survey sought to gain insights into people’s perspectives on self-sampled HPV diagnostic tests, including: desirable design features that may facilitate using these diagnostic tests; and identifying sociodemographic factors that may contribute to inequalities in peoples’ ability to use the tests, based on PROGRESS-Plus (Place of residence, Race/ethnicity/culture/language, Occupation, Gender/sex, Religion, Education, Socioeconomic status, Social capital, Personal characteristics, Time-dependent relationships, Features of relationships). PROGRESS-Plus is an established framework used to identify factors that can stratify and impact health opportunities and outcomes and was used because it provides a systematic and widely recognized approach to identifying sociodemographic associated with health inequalities and/or inequities (17;18). The survey included multiple-choice questions with additional open-ended questions and was completed anonymously, with only some sociodemographic information collected. At the end of the survey, respondents were provided the opportunity to express an interest in attending one of the focus groups. The survey was piloted internally within the research team to check for inaccuracies and to test the survey logic.

#### Distribution

The opportunity to participate in the survey was advertised through the NIHR Innovation Observatory’s social media channels (X and LinkedIn) and directly emailed to specific relevant contacts, including cervical cancer and women’s health charities and professional bodies based in the UK. Snowball sampling was employed, where respondents were asked to help connect us with other interested individuals. The survey remained open from 20 August 2024 until 5 September 2024.

#### Data analysis

Survey responses were collated on Qualtrics and downloaded into Excel for cleaning and analysis. All responses were anonymized and descriptive analyses of responses were conducted, with comparisons made between different groups of respondents. Responses relating to design features and PROGRESS-Plus domains were stratified by respondent role (i.e., member of the public, clinician, researcher, or other) to understand the influence of this.

#### Focus group methods

Separate online focus groups were conducted with clinicians and members of the public to discuss the main survey findings and potential equity considerations in greater detail. An online format was chosen to enable participants to join from different geographical locations across the UK. A purposive sampling strategy using demographic information (e.g., age group, ethnic group) was employed to select a broader range of participants from those survey respondents who expressed an interest in participating. Those who were purposively sampled were sent a consent form to participate via Qualtrics; copies of the consent forms can be found in Supplementary Materials 3 and 4.

#### Design and facilitation

The design of the focus groups was adapted from a framework developed to elicit perspectives on equity factors that may affect people’s ability to adopt or use a specific intervention (19). Both groups were held using Zoom and were scheduled to last for one hour. The topic guides were designed to elicit responses surrounding: the perceived acceptability of using self-sampled diagnostic tests for HPV; potential barriers to their use; the relative importance of the different barriers identified; and whether perspectives would change if all potential barriers to adoption were removed. Initial prompts were derived from the results of the stakeholder survey and what was considered most important to each group. A copy of the topic guides can be found in Supplementary Material 5.

One researcher (SG) led each focus group, with another (either BH or IF) monitoring the Zoom chat log, assisting with timekeeping, and addressing any technical issues, if needed. Each focus group was recorded via Zoom, with two researchers (BH or IF) taking additional notes to supplement the recording.

### Data analysis

Recordings, automated Zoom transcripts, and chat logs from both focus groups were downloaded and securely stored in password-protected files on Microsoft Teams, accessible only to the research team. Basic demographic information of participants (age group, ethnic group) provided in the survey was tabulated. One researcher (EEJ) checked transcripts downloaded from Zoom for accuracy against the recording, anonymizing participant names prior to analysis.

Deductive analysis using a framework based on the domains required for the horizon scan, including design principles and PROGRESS-Plus domains, was conducted (17;18). Emerging concepts could be inductively coded, if required. Two researchers (EEJ and SG) coded each focus group transcript based on the framework and added any codes that may potentially have been missed or added queries to ensure no relevant data were erroneously excluded. Any differences were resolved through discussion between the researchers. After agreeing on the final codes, the number of specific design principles, PROGRESS-Plus domains, and other emerging themes mentioned in each group were counted and tabulated (17;18). Commonalities and differences between the findings from the two groups were identified using a brief narrative approach.

### Survey results

#### Characteristics of survey respondents

**Figure 1.**
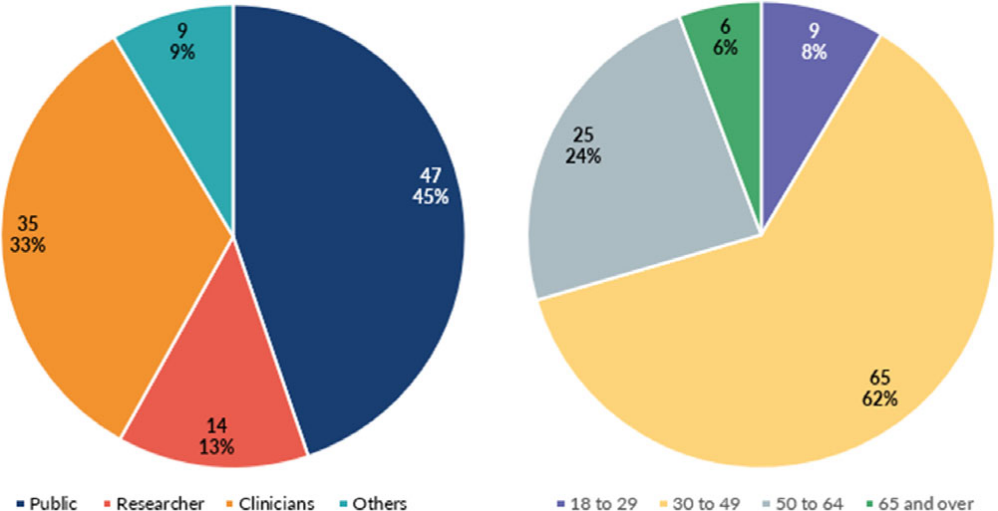
Role and age ranges of survey respondents.

Figure 1 shows the age ranges and roles of people who responded to the survey. In total, 105 of 111 individuals who took part in the survey fully completed the questionnaire. Nearly 45 percent of participants (*n* = 47) identified as members of the public, around 33 percent (*n* = 35) as clinicians, and 13 percent (*n* = 14) as researchers. A further 8.5 percent (*n* = 9) identified as “other.” Ninety-three percent of participants were White British (*n* = 98). Most participants were aged 30–49 (*n* = 65; 62 percent). Around 68 percent of participants (*n* = 71) had no previous knowledge of HPV home tests. Of these, 40 percent (*n* = 14) of clinicians and 85 per cent of members of the public (*n* = 40) reported no prior knowledge of HPV self-testing.

#### Design features

**Figure 2.**
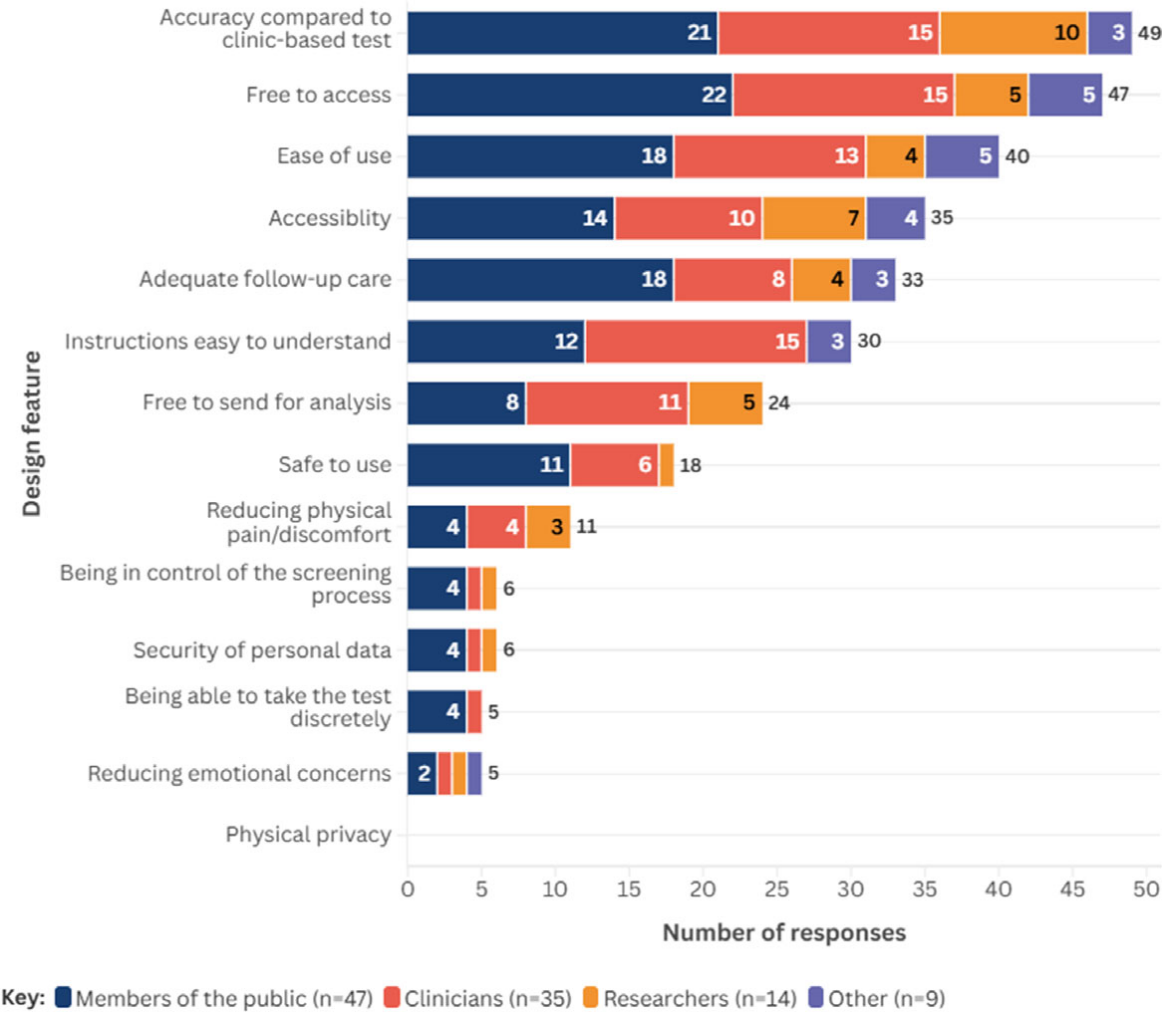
Design features considered most important to respondents, stratified by role.

Across all participants, the five most important design features of self-sampled HPV tests were: similar accuracy as when performed by a clinician (~47 percent, *n* = 49); free access to a self-sampled HPV test (~45 percent, *n* = 47); ease of use (~38 percent, *n* = 40); accessibility (~33 percent, *n* = 35); and adequate follow-up (~31 percent, *n* = 33). Design features considered less important were: reducing emotional concerns such as embarrassment (~5 percent, *n* = 5); and the ability to take the test discreetly (~5 percent, *n* = 5). No respondents suggested physical privacy as an important design principle. Figure 2 presents the design features considered most important by people who undertook the survey, stratified by role; further information on results stratified by role is presented in Supplementary Material 6.

#### PROGRESS-Plus domains

**Figure 3.**
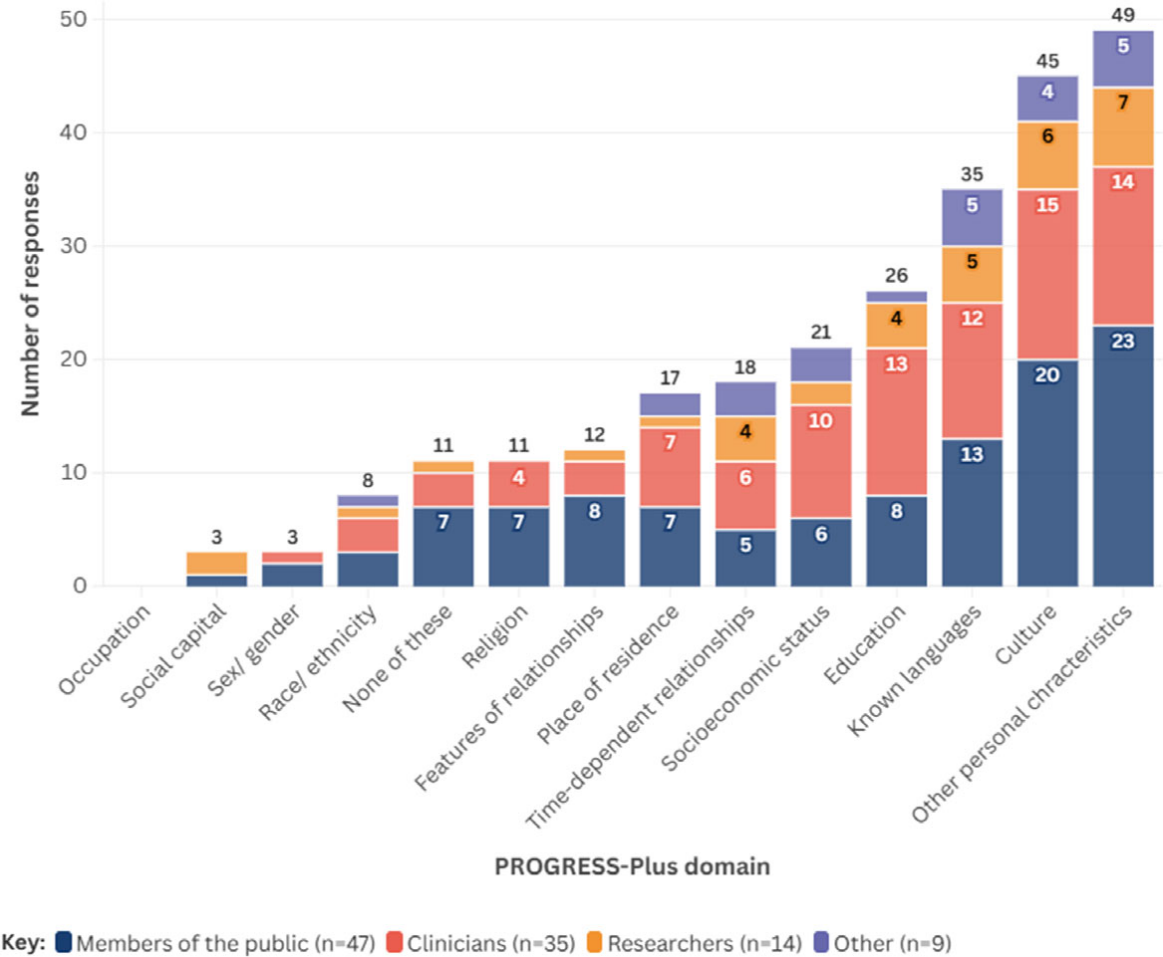
PROGRESS-Plus domains considered important by survey respondents, stratified by role.

Around 47 percent of all respondents (*n* = 49) suggested that personal characteristics (e.g., age, having a disability) may have the biggest impact on people’s ability to use a self-sampled HPV test. Culture was also noted by around 43 percent of participants (*n* = 45), followed by language(s) spoken (~33 percent, *n* = 35), and level of education (25 percent, *n* = 26). The least likely factors perceived to impact people’s ability to use a self-sampled HPV test were level of social capital (~3 percent, *n* = 3) and sex/gender (~3 percent, *n* = 3). No respondents suggested that an individual’s occupation would have an impact on their ability to use or access a self-sampled test for HPV. Figure 3 shows the PROGRESS-Plus factors suggested by survey respondents to have the potential to affect accessibility, stratified by respondent group; further information on results stratified by role is presented in Supplementary Material 6. Twenty respondents provided additional perspectives as free text within the survey; these are detailed within Supplementary Material 7.

## Focus group results

### Characteristics of participants

Five members of the public attended one focus group, whereas four clinicians attended the second; demographics are summarized in Table 1. Of the nine people who attended, most were either aged between 50 and 64 or were 65 and over (*n* = 3, 33 percent each), whereas eight participants were White (88 percent).

**Table 1.** Summary of demographics of focus group participants

	Members of the public (*n* = 5)	Clinicians (*n* = 4)	Total (*n* = 9)
*Age group*			
18–29	1 (20%)	0	1 (12%)
30–49	1 (20%)	1 (25%)	2 (22%)
50–64	1 (20%)	2 (50%)	3 (33%)
65 and over	2 (40%)	1 (25%)	3 (33%)
Prefer not to say	0	0	0
*Ethnic group*			
White	4 (80%)	4 (100%)	8 (88%)
Asian/Asian British	1 (20%)	0	1 (12%)
Black/African/ Caribbean/Black British	0	0	0
Chinese	0	0	0
Arab	0	0	0
Other ethnic groups	0	0	0
Prefer not to say	0	0	0

*n*, number of participants.

### Design principles

#### Support and follow-up

Both clinicians and members of the public emphasized the need for adequate follow-up care and a clear pathway for people with positive test results. Clinicians noted that, ideally, this should be established at a national level. Clinicians discussed this from an organizational and service delivery perspective, highlighting that, without a clear pathway, patients may be left uncertain about their next steps, especially if private companies provide tests without integrated healthcare support. They discussed other challenges not raised by members of the public, such as legal responsibilities and patient management, particularly if private companies administered tests without sufficient follow-up support.

Given that some cervical screening visits offer opportunities for broader health guidance (e.g., on smoking cessation or contraception), clinicians suggested that the potential for essential public health advice and other forms of opportunistic contact typically provided during in-clinic appointments would be absent in a home-testing model.

*What I’d be concerned about sometimes is people become very like: Oh, your HPV test was negative. It’s fine. You don’t need to be seen. So, if someone’s got, you know, vaginal or unexpected bleeding, the literature needs to be very clear about having a negative HPV result doesn’t mean that you don’t go and seek help if you’ve got symptoms. So yeah, I think the literature would need to be very clear about that.* - Clinician.

Furthermore, some clinicians questioned how HPV testing would integrate with existing HPV vaccination programs, debating whether vaccinated individuals would still need regular screening and how frequently testing should occur for different risk groups.

Members of the public discussed impact at the individual level more, expressing a desire for immediate support following a positive test. One participant highlighted that, if results arrived via post, some individuals may be left feeling isolated or anxious about what a positive test result could mean. To mitigate potential issues surrounding privacy and follow-up, participants proposed different options to delay or customize contact preferences upon receiving results, potentially helping individuals manage the emotional impact privately.

*it worries me that you get sent the result through the post and you could be on your own and thinking; oh. I’ve got cancer, and I’ve only got a little times left. So, if I had a positive [test] that would freak me out, so I think it should be the results for the doctor, if it’s a positive result* – Member of the public.

#### Convenience and accessibility

Both clinicians and members of the public suggested that self-sampled HPV testing could improve convenience, particularly for busy individuals or those uncomfortable with in-clinic testing. Clinicians suggested the approach may reduce physical pain and discomfort, offering a discreet alternative to clinic testing, whereas members of the public highlighted how being able to take the test at home would reduce the need to visit multiple locations, noting the comfort and privacy of self-sampling.

*So, if we can make testing easier, so they don’t have to contact a GP. They don’t need to come to me and sort of say, well, you know, I think I might have it, because they can just do it, and then take it from there… and especially for people with conditions like vaginismus. They have a lot more control […] They’re probably more likely to do that at home than come in for a smear.* - Clinician.

*It’s more convenient, isn’t it? You’re not having to go running around to different places to go and get tested. Just do it in the comfort of your own home and send it off* – Member of the public.

#### Affordability

The costs of self-sampling were discussed by both groups. Clinicians noted that, if individuals are required to fund the test themselves, this could discourage usage, particularly among lower-income groups. This was echoed by members of the public, who suggested that providing free tests would encourage greater uptake.

#### Other concepts

Other concepts relating to design principles included privacy, autonomy, clarity, and ease of use. Further discussion is presented in Supplementary Material 8.

### Equity considerations

#### Occupation, socio-economic status, and features of relationships

There were differences in how important participants from each group believed different factors were. Clinicians discussed the specific benefits and considerations of offering self-sampled HPV testing for groups that may be underserved by traditional cervical screening methods, suggesting several groups who might benefit, including: sex workers; individuals who have experienced sexual trauma; those who experience conditions like vaginismus; and trans men.

*… I think for any women who have had kind of sexual trauma, or experience vaginismus, or any kind of other issues while been examined when using a speculum is definitely good […] people who are sort of perimenopausal, postmenopausal, because then you know, from that 50 to sort of 60 mark where they still need a smear, but it may be more difficult for them.* - Clinician.

Clinicians working with vulnerable populations, including sex workers and homeless individuals, expressed that easier access to HPV testing could enable outreach and regular screening for these groups.

Clinicians highlighted that self-sampled HPV tests could help address barriers faced by individuals with limited autonomy over their healthcare, including those experiencing domestic abuse. In such contexts, accessing care discreetly was considered crucial. However, due to the sexually transmitted nature of HPV, a positive result could create safety risks for individuals – particularly women – in abusive relationships, where such health information might be used to exert control or cause harm. These concerns were echoed by members of the public, who noted that the visibility of test kits or results could pose risks if discovered by family members or partners. Results arriving by post or being accessed through shared or controlled digital devices were seen as a potential threat to confidentiality and personal safety.

#### Race/ethnicity, culture, and language

Members of the public placed greater emphasis on cultural factors and religious beliefs as potential barriers to accessing and using self-sampled HPV tests than clinicians, particularly where premarital sexual activity may be discouraged or considered taboo. These perspectives highlight how, whereas self-sampled tests are often promoted to enhance privacy and autonomy, in certain cultural or household contexts they may paradoxically increase concerns surrounding visibility, stigma and unintended disclosure. Referring to intergenerational living arrangements, one member of the public noted:

*… and quite a few of them live together and you wouldn’t want that letter [test results] to arrive in the post because someone could see it and wonder what’s going on.*

Clinicians mentioned how, in some ethnic minority communities, cultural beliefs around sexual health might limit engagement. One clinician highlighted barriers faced by isolated ethnic minority women, particularly those who do not speak English, with language identified as a primary barrier to accessing support. Clinicians also suggested that specific groups (such as individuals who do not speak English or have limited healthcare access) might remain underserved even if self-sampled testing options were available, noting the need for targeted approaches for such populations.

#### Community engagement and time-dependent factors

Community engagement and time-dependent factors were identified as another concept emerging from the focus groups. Further discussion is presented in Supplementary Material 8.

## Other concepts

Participants in both focus groups discussed concepts related to self-sampled HPV tests unrelated to design or accessibility; these are described in Supplementary Material 6.

### Discussion

The findings of this mixed-methods study highlight potential barriers and facilitators to the use of self-sampled HPV tests, particularly from the perspectives of clinicians and members of the public. The survey findings (*n* = 105) provided quantitative insights into key priorities, whereas the qualitative focus groups facilitated in-depth discussions regarding the acceptability and accessibility of self-sampled HPV tests, revealing both shared and divergent priorities.

Participants in both the survey and focus groups identified accessibility, ease of use, and clear follow-up pathways as being essential to successful self-sampled HPV testing, emphasizing the importance of offering free tests to ensure equitable access. Participants in these groups also highlighted the privacy and comfort offered by self-sampling, noting its potential to alleviate logistical challenges and emotional discomfort, particularly for underserved or vulnerable populations. These findings suggest that self-sampled HPV testing could help reduce existing health inequalities by improving privacy, comfort and convenience and by addressing some logistical barriers to screening. However, there is a possibility it could also unintentionally reinforce inequalities if barriers related to cost, access, clear follow-up pathways, stigma, and cultural or relationship factors are not addressed.

The survey and focus groups diverged slightly in their emphasis on specific populations and relational dynamics. The focus groups identified sex workers and individuals affected by domestic abuse as groups particularly likely to benefit from self-sampled testing. Both methods highlighted cultural and social factors as potential barriers to accessing and using self-sampled HPV tests. In line with previous evidence (10;20), participants in this study viewed self-sampled HPV testing as potentially acceptable and beneficial, particularly because of increased privacy, comfort, and convenience. However, our findings place greater emphasis on the conditions required for equitable implementation, particularly free access and clear follow-up pathways. In addition, the focus groups drew attention to relationship and social-context barriers (including abuse, stigma, and religious beliefs), which are less explicitly foregrounded in some acceptability-focused studies. This suggests that future research should evaluate not only uptake and acceptability, but also differential impacts across population groups and the effectiveness of implementation supports.

#### Strengths and limitations

This work has several strengths, enhancing the reliability of its findings. We sent the stakeholder survey to a wide variety of organizations, charities, and networks with an interest in the topic and received a total of 105 completed responses. The responses were from different stakeholder groups, with 45 percent being members of the public, 33 percent clinicians, 13 percent researchers, and 8.5 percent from another role. We used the results of the survey to develop prompts and key questions for the two focus groups, ensuring that we were able to tailor the questions according to what members of the public and clinicians deemed most important in the survey.

However, due to the small size of the focus groups, perspectives on the topic could only be reflected partially. In particular, the study may not fully capture the views of public health practitioners, specialist clinicians, and people who may not be interested in self-sampling for HPV or who are hesitant to be screened. Additionally, participants were predominantly white, with limited representation of minority ethnic communities. Other groups, including individuals with disabilities, LGBTQ+ communities, and sex workers, were also underrepresented. This under-representation may limit the generalizability of the findings.

Furthermore, we took a largely deductive and descriptive approach to analyzing the focus group data, using a predefined framework. We considered this approach appropriate for the purpose of the focus groups in the context of the wider work, where the results would be used to inform and contextualize the results of a horizon scan. However, we may have identified further themes had a different approach been adopted (e.g., thematic analysis).

#### Implications for further research and practice

From a healthcare system perspective, clinicians at our focus groups highlighted the need to ensure that self-sampled tests for HPV are adequately embedded into the current clinical care pathway for cervical screening, including clear onward pathways for people with positive diagnoses to avoid leaving people unsupported (21). A public consultation on a recommendation that self-sampling should be offered to under-screened individuals within the cervical screening program was opened in December 2024 and closed on 26 February 2025 (22). Furthermore, the National Institute for Health and Care Research is commissioning an in-service evaluation on the routine use of self-sampling in the NHS Cervical Screening Program to determine how well the approach would work in practice and what the implications would be if self-sampling were offered as an optional screening method (23). These consultations may enhance clarity surrounding how self-sampling for HPV could fit into the wider cervical screening program.

Our findings suggest that any future research surrounding the acceptability and diagnostic accuracy of self-sampling tests for HPV should assess factors considered important to end users, such as ease of use, clarity of instructions, test accuracy, and the level of pain and discomfort experienced. Such evaluations should also ensure that different groups are involved in the assessment, including a diversity of age groups, people from different cultures, who speak different languages, and have different educational levels and socioeconomic backgrounds. As highlighted by our focus groups, the participation of groups who may otherwise be underserved by current in-clinic cervical screening methods should be encouraged, such as sex workers, trans men, people who have experienced sexual trauma, homeless people, and those who experience conditions such as vaginismus. As suggested by Choi et al (2023), developing messaging around HPV screening could be tailored to specific subgroups, helping to address different concerns that impact attendance at clinic-based screening (24).

The findings of the survey and focus groups fed into the design and interpretation of a linked horizon scan investigating emerging self-sampling diagnostic tests for HPV, informing the prioritization of design principles, adding key design features to extract and helping to contextualize the results; this will be detailed in a future publication. Future horizon scanning may wish to consider embedding similar mixed-methods to inform their overall design, ensuring that such research is responsive to the needs of potential end users.

## Conclusions

This study highlighted potential considerations that may have an influence on effectively implementing self-sampled HPV tests. Diagnostic accuracy, ease of use, cost-free access, clear instructions, accessibility, and robust follow-up pathways were identified as important to successful adoption. Clinicians and the public in focus groups also identified potential barriers to access and use, including disability, language, cultural attitudes, and socioeconomic status. To address these issues, policymakers and healthcare providers should prioritize different communities’ unmet needs and develop clear clinical pathways for providing self-sampling tests and managing their results. Effectively integrating self-sampled HPV tests into existing screening programs has the potential to significantly enhance accessibility, reduce health disparities, and contribute to the broader goal of cervical cancer elimination.

## Supporting information

10.1017/S0266462326103729.sm001 Ghasri et al. supplementary material Ghasri et al. supplementary material

## Supplementary material

The supplementary material for this article can be found at http://doi.org/10.1017/S0266462326103729.
