# Supplementary material for: Barriers and facilitators to using self-sampled tests for the human papillomavirus (HPV): a mixed-methods study to inform a horizon scan
Source: Int J Technol Assess Health Care. 2026 Apr 13;42(1):e46. doi: 10.1017/S0266462326103729 (PMC13161927; doi:10.1017/S0266462326103729)
Supplement: Ghasri et al. supplementary material [file S0266462326103729sup001.docx]

**Supplementary Material 1: Good Reporting of A Mixed Methods Study (GRAMMS) checklist**

| **Item number** | **Guidance** | **Page number** |
| --- | --- | --- |
| 1 | Describe the justification for using a mixed methods approach to the research question | 3-4 |
| 2 | Describe the design in terms of the purpose, priority and sequence of methods | 4-6 |
| 3 | Describe each method in terms of sampling, data collection and analysis | 4-6 |
| 4 | Describe where integration has occurred, how it has occurred and who has participated in it | 5 |
| 5 | Describe any limitation of one method associated with the present of the other method | 14 |
| 6 | Describe any insights gained from mixing or integrating methods | 15 |

**Supplementary Material 2: Copy of stakeholder survey**

**Help us with our research to understand more about adopting tests that can be used at home to detect HPV**

**Thank you for your interest in completing this survey as part of our research about diagnostic tests for the human papillomavirus (HPV) that could be used at home.**

**Who are we looking for?**

We are looking for clinicians, members of the public and anyone with an interest in testing for human papillomavirus (HPV) to complete this survey. You must be 18 or older to compete this survey.

**What does this project involve?**

We are doing a horizon scan to find what diagnostic tests for HPV that can be used outside of a clinical setting (e.g. a doctor's office) are ready to come on to the market. Horizon scanning is a method used by researchers to find out what types of health technologies (such as medicines and diagnostic tests) could potentially come into use within the health service in the future. This information can then be used to help inform policymakers in advance.

To help inform this horizon scan, we want to know more about what people think how tests for HPV at home should be designed and what might help or stop people from using them. This survey is the first part of this process and will help us to know more about what features to look out for when we are looking for tests relevant to our horizon scan.

**What will taking part in this survey involve?**

We anticipate that it will take around 5 to 10 minutes to complete this survey.

We will ask you to provide some demographic information (such as your age group and your ethnicity) so that we can understand more about whether different groups have different perspectives and preferences. However, you will also be given the option not to disclose this information. We will then ask you to answer some multiple-choice questions and there will be an opportunity to add any additional views you may have at the end of these questions.

We are also going to hold two focus groups as part of this project to discuss these issues in more detail in small groups. At the end of this survey, you will be asked if you would be interested in attending one of these focus groups. If you are interested, you will be asked to provide your name and email address.

**Has this study received ethical approval?**

This study was approved by the Faculty of Medical Sciences Research Ethics Committee, part of Newcastle University’s Research Ethics Committee. This Committee includes members who are internal to the faculty. This study was reviewed by members of the Committee, who must provide impartial advice and avoid significant conflicts of interests.

**What data will we collect and store?**

We will use some information you provided in the survey (e.g. age group and ethnicity) within our report. If you are interested in attending one of our focus groups, we will ask you for your name and email address. We will store all the data collected in this survey securely on an encrypted Newcastle University platform and will not share the data with anyone beyond those involved in conducting the research.

Newcastle University will act as the data controller for this study, ensuring your information is used appropriately and in the public interest. You can find out more about this here <https://www.ncl.ac.uk/data.protection/dataprotectionpolicy/privacynotice/> or by contacting Newcastle University's Data Protection Officer (Maureen Wilkinson, [rec-man@ncl.ac.uk](mailto:rec-man@ncl.ac.uk)).

**Are there any risks in taking part in this survey?**

Although we will not be asking you to disclose any personal experiences of HPV testing in this survey, we recognise this is a sensitive topic area. Further information on HPV, including testing, can be found on the NHS website (embed: https://www.nhs.uk/conditions/human-papilloma-virus-hpv/) and via [Gov.uk](https://www.gov.uk/government/publications/cervical-screening-support-for-people-who-find-it-hard-to-attend/cervical-screening-support-for-people-who-feel-anxious-about-attending) (embed: https://www.gov.uk/government/publications/cervical-screening-support-for-people-who-find-it-hard-to-attend/cervical-screening-support-for-people-who-feel-anxious-about-attending).

**Can I stop taking part?**

Your participation is voluntary. You have the right not to answer any question that you do not wish to. You have the right to withdraw your data for any reason after completing the survey by emailing insights@io.nihr.ac.uk.

**Who do I contact if I have a concern about the research, or I wish to complain?**

If you have a concern or any queries about any aspect of this study, please contact: [research.integrity@ncl.ac.uk](mailto:research.integrity@ncl.ac.uk)

[Section break]

**Participant consent**

**By taking part in this survey, you are providing your consent to participate in this study. Please confirm your consent by checking the boxes below:**

- I confirm that I have read and understood the previous information and agree to participate in this survey
- I consent to the dissemination and publication of my responses as part of the research data
- I confirm that I am 18 years or older

Please provide the first three letters of your surname below to help us identify and remove your data if you choose to withdraw from the study.

[Section break – Survey will end here if ‘No’ is selected for any of the above. All above are mandatory fields]

**Q1: How would you best describe yourself?**

- Clinician
- Member of the public
- Researcher
- Policymaker
- Prefer not to say
- Other (please describe)

**Q2. What is your age range?**

- 18 to 29
- 30 to 49
- 50 to 64
- 64 and over
- Prefer not to say

**Q3: What is your ethnic group?**

- White
- Asian/Asian British
- Black/African/Caribbean/Black British
- Chinese
- Arab
- Other ethnic group
- Prefer not to say

[Section break]

**Q4. Before taking part in this survey, had you heard of HPV test kits that could be used at home?**

- Yes
- No

**Q5. Which of these features do you think are important when considering whether to use a HPV test at home?**

[Participants will be asked whether these are “Important” or “Not important”]

- If the test was free to access
- If it was free to send the test away for analysis
- Accessibility (e.g. no need to go to a GP to be screened)
- Ease of use
- Instructions that are easy to understand
- Safe to use
- Physical privacy
- Being able to take the test discretely
- Knowing that your personal data was secure
- Reducing emotional concerns (e.g. anxiety or embarrassment around screening)
- Reducing physical pain or discomfort of screening
- Feeling more like you are in control of the screening process
- Knowing that the test is as accurate as when performed by a clinician
- Having adequate follow-up care (e.g. being able to see a clinician quickly if a test was positive)

**Q6. Of these features, which three would be most important to you?**

[Participants will be asked to select 3 options]

- If the test was free to access
- If it was free to send the test away for analysis
- Accessibility (e.g. no need to go to a GP to be screened)
- Ease of use
- Instructions that are easy to understand
- Safe to use
- Physical privacy
- Being able to take the test discretely
- Knowing that your personal data was secure
- Reducing emotional concerns (e.g. anxiety or embarrassment around screening)
- Reducing physical pain or discomfort of screening
- Feeling more like you are in control of the screening process
- Knowing that the test is as accurate as when performed by a clinician
- Having adequate follow-up care (e.g. being able to see a clinician quickly if a test was positive)

[Section break]

**Q7. Do you think any of the following personal characteristics might have an effect on someone’s ability or opportunity to use a HPV test at home? Tick up to three that you think might have an effect.**

- Where a person lives
- Their race and ethnicity
- Their culture
- The languages they know
- Someone’s occupation
- Someone’s sex or gender
- Their religion
- Their level of education
- Their socioeconomic status (such as their level of income)
- Their social capital (such as their relationship with other people in the community)
- Other personal characteristics (such as if they have a disability)
- Features of their relationships (such as their relationship with other people they live with)
- Any other time-dependent relationship (such as if the person has just left hospital or is at a temporary disadvantage)
- None of these

[Section break]

**Q8. Please use this space to tell us about any features of diagnostic tests for HPV that can be used at home we have missed that you would like us to consider further.**

[Open ended question]

[Section break]

**Q9. Thank you for participating in this survey. We will be hosting two focus groups in [Month TBC] to discuss what might help or prevent people from doing HPV testing at home in more detail as part of a small group.**

**Would you be interested in attending one of these focus groups?**

- Yes
- No

[Survey will end here if ‘No’ is selected, with the following message: Thank you for completing this survey.

Further information on HPV, including testing, can be found on the NHS website (embed: https://www.nhs.uk/conditions/human-papilloma-virus-hpv/) and via [Gov.uk](https://www.gov.uk/government/publications/cervical-screening-support-for-people-who-find-it-hard-to-attend/cervical-screening-support-for-people-who-feel-anxious-about-attending) (embed: https://www.gov.uk/government/publications/cervical-screening-support-for-people-who-find-it-hard-to-attend/cervical-screening-support-for-people-who-feel-anxious-about-attending).

[If ‘Yes is selected for the above question, the following message will display upon submission]

**Q10. Thank you for your interest in attending one of our focus groups. So that we can contact you with further details, please provide your name and email address.**

Your name:

Your email address:

[Survey will end here if with the following message]

Thank you for completing this survey and for expressing an interest in joining one of our focus groups. We will email you with further information in due course.

Further information on HPV, including testing, can be found on the NHS website (embed: https://www.nhs.uk/conditions/human-papilloma-virus-hpv/) and via [Gov.uk](https://www.gov.uk/government/publications/cervical-screening-support-for-people-who-find-it-hard-to-attend/cervical-screening-support-for-people-who-feel-anxious-about-attending) (embed: https://www.gov.uk/government/publications/cervical-screening-support-for-people-who-find-it-hard-to-attend/cervical-screening-support-for-people-who-feel-anxious-about-attending).

**Supplementary Material 3: Consent form for focus group with clinicians**

**Help us with our research to understand more about adopting tests that can be used at home to detect HPV**

**Who are we looking for?**

You have previously indicated that you would be interested in joining a focus group to further discuss diagnostic tests for the human papillomavirus (HPV) that can potentially be used at home.

Before deciding to take part, it is important you understand why the research is being done and what it will involve. Please read the following carefully and discuss it with others if you wish.

If anything is not clear or you would like more information, please email: insights@io.nihr.ac.uk

**Why are we doing these focus groups?**

We want to build on the results of our previous survey and discuss diagnostic tests for HPV that can be used at home in more detail. We are holding two focus groups, one with clinicians and one with members of the public, to understand more about what might help or stop people from using these tests. We are hoping to share the findings as a report and an academic paper.

The focus group with clinicians will take place on Wednesday 16th October between 6-7pm.

**What will taking part in a focus group involve?**

We will be holding two English language focus groups with up to seven other people, conducted and recorded on Microsoft Teams or Zoom. In the focus group, you will be given a short summary of our work to date and we will then ask the focus groups to discuss potential adoption of HPV testing kits to use at home, including discussing different factors that might help or prevent people using these tests. We will not ask you for any personally identifying information during the focus group and we will not ask any questions about your personal experiences of HPV testing.

While we will not ask you to share personal experiences of HPV testing, our discussions may touch on sensitive topics such as the use of vaginal and cervical swabs, urine sampling, anal or penile testing for HPV, and factors that might discourage participation in HPV testing.

**Has this study received ethical approval?**

This study was approved by the Faculty of Medical Sciences Research Ethics Committee, part of Newcastle University’s Research Ethics Committee. This Committee includes members who are internal to the Faculty. This study was reviewed by members of the Committee, who must provide impartial advice and avoid significant conflicts of interests.

**What data will we collect and store?**

We will ask you to provide your name as an indication of consent to take part and your e-mail to allow us to contact you about outcomes of the study. We will keep this data separate to any other data collected.

We will use some information you provided in the survey (e.g. age group and ethnicity) within our report but we will not be collecting any other personally identifying information during the focus group. You will not be personally identifiable in any reports or publications about the project.

We will record the focus groups to make sure we are accurately capturing the discussions. We will transcribe (type up) these recordings, which will be fully anonymised, and then delete the recordings. We will securely store all data collected using a Newcastle University encrypted platform, which only the researchers involved in the project will have access to. We will only disclose information you provide if there is a legal requirement to do so (for example, under statute or a court order) and/or we have an overriding duty to the public (for example, the information concerns the commission of a criminal offence or relates to life-threatening circumstances).

Newcastle University will act as the data controller for this study, ensuring your information is used appropriately and in the public interest. You can find out more about this here or by contacting Newcastle University's Data Protection Officer (Maureen Wilkinson, rec-man@ncl.ac.uk).

**Are there any risks in taking part?**

Taking part in this project will lead to a better understanding of what may help or stop people testing for HPV within their own homes. Although we will not be asking you to disclose any personal experiences of HPV testing, we recognise this is a sensitive topic area. If you experience distress during the focus group, you can pause or stop your participation as needed, or you may withdraw from the focus group.

**Can I stop taking part?**

If you do decide to take part, you can stop at any point without a reason just by letting the researcher know. If you stop after the recording of the focus group has been transcribed or anonymised, your consent form and email address will be deleted but we will be unable to identify your interview data for removal.

**Who do I contact if I have a concern about the research, or I wish to complain?**

If you have a concern or any queries about any aspect of this study, please contact research.integrity@ncl.ac.uk

End of Block: Default Question Block

Start of Block: Block 1

Q6 **Consent form**

Please complete the following consent form while considering the information you have read above and/or subsequently discussed. Please tick the appropriate boxes.

**Taking part in the study**

I confirm that I have read the information sheet provided on [DATE], had the opportunity to consider the information, had the opportunity to ask questions and have had any questions answered satisfactorily.

- Yes (1)
- No (2)

Q7 I understand that my participation is voluntary and that I can stop at any time. I understand that if I stop, any data I have provided up to that point (as far as is plausible) will be deleted.

- Yes (1)
- No (2)

Q8 I understand how my personal information will be used for this study.

- Yes (1)
- No (2)

Q9 I understand that I will be recorded, that this will be stored anonymously on password-protected software, used for research purposes only, then destroyed after completion of the transcription.

- Yes (1)
- No (2)

Q10 **How my information will be used after the project**

I understand that personal details, such as my name and email address, will not be revealed to anyone outside of the project.

- Yes (1)
- No (2)

Q11 I am happy for my anonymized, transcribed data to be stored and used by others for future research.

- Yes (1)
- No (2)

Q12 I understand and agree my data may be published as a journal article and may appear in other materials used to share the findings of the project.

- Yes (1)
- No (2)

End of Block: Block 1

Start of Block: Block 2

Q14 Please confirm that you agree (consent) to take part in this research project by signing below.

- Name (1) __________________________________________________
- Email address (2) __________________________________________________
- Date of completion (3) __________________________________________________

End of Block: Block 2

**Supplementary Material 3: Consent form for focus group with members of the public**

**Help us with our research to understand more about adopting tests that can be used at home to detect HPV**

**Who are we looking for?**

You have previously indicated that you would be interested in joining a focus group to further discuss diagnostic tests for the human papillomavirus (HPV) that can potentially be used at home.

Before deciding to take part, it is important you understand why the research is being done and what it will involve. Please read the following carefully and discuss it with others if you wish.

If anything is not clear or you would like more information, please email: insights@io.nihr.ac.uk

**Why are we doing these focus groups?**

We want to build on the results of our previous survey and discuss diagnostic tests for HPV that can be used at home in more detail. We are holding two focus groups, one with clinicians and one with members of the public, to understand more about what might help or stop people from using these tests. We are hoping to share the findings as a report and an academic paper.

The focus group with members of the public will take place on Monday 14th October between 6-7pm.

**What will taking part in a focus group involve?**

We will be holding two English language focus groups with up to seven other people, conducted and recorded on Microsoft Teams or Zoom. In the focus group, you will be given a short summary of our work to date and we will then ask the focus groups to discuss potential adoption of HPV testing kits to use at home, including discussing different factors that might help or prevent people using these tests. We will not ask you for any personally identifying information during the focus group and we will not ask any questions about your personal experiences of HPV testing. While we will not ask you to share personal experiences of HPV testing, our discussions may touch on sensitive topics such as the use of vaginal and cervical swabs, urine sampling, anal or penile testing for HPV, and factors that might discourage participation in HPV testing.

For your time, you will receive a £25 digital gift voucher.

**Has this study received ethical approval?**

This study was approved by the Faculty of Medical Sciences Research Ethics Committee, part of Newcastle University’s Research Ethics Committee. This Committee includes members who are internal to the Faculty. This study was reviewed by members of the Committee, who must provide impartial advice and avoid significant conflicts of interests.

**What data will we collect and store?**

We will ask you to provide your name as an indication of consent to take part and your e-mail to allow us to contact you about outcomes of the study. We will keep this data separate to any other data collected.

We will use some information you provided in the survey (e.g. age group and ethnicity) within our report but we will not be collecting any other personally identifying information during the focus group. You will not be personally identifiable in any reports or publications about the project.

We will record the focus groups to make sure we are accurately capturing the discussions. We will transcribe (type up) these recordings, which will be fully anonymised, and then delete the recordings. We will securely store all data collected using a Newcastle University encrypted platform, which only the researchers involved in the project will have access to. We will only disclose information you provide if there is a legal requirement to do so (for example, under statute or a court order) and/or we have an overriding duty to the public (for example, the information concerns the commission of a criminal offence or relates to life-threatening circumstances).

Newcastle University will act as the data controller for this study, ensuring your information is used appropriately and in the public interest. You can find out more about this here or by contacting Newcastle University's Data Protection Officer (Maureen Wilkinson, rec-man@ncl.ac.uk).

**Are there any risks in taking part?**

Taking part in this project will lead to a better understanding of what may help or stop people testing for HPV within their own homes. Although we will not be asking you to disclose any personal experiences of HPV testing, we recognise this is a sensitive topic area. If you experience distress during the focus group, you can pause or stop your participation as needed, or you may withdraw from the focus group.

**Can I stop taking part?**

If you do decide to take part, you can stop at any point without a reason just by letting the researcher know. If you stop after the recording of the focus group has been transcribed or anonymised, your consent form and email address will be deleted but we will be unable to identify your interview data for removal.

**Who do I contact if I have a concern about the research, or I wish to complain?**

If you have a concern or any queries about any aspect of this study, please contact research.integrity@ncl.ac.uk

End of Block: Default Question Block

Start of Block: Block 1

**Consent form**

Please complete the following consent form while considering the information you have read above and/or subsequently discussed. Please tick the appropriate boxes.

**Taking part in the study**

I confirm that I have read the information sheet provided on [DATE], had the opportunity to consider the information, had the opportunity to ask questions and have had any questions answered satisfactorily.

- Yes (1)
- No (2)

I understand that my participation is voluntary and that I can stop at any time. I understand that if I stop, any data I have provided up to that point (as far as is plausible) will be deleted.

- Yes (1)
- No (2)

I understand how my personal information will be used for this study.

- Yes (1)
- No (2)

I understand that I will be recorded, that this will be stored anonymously on password-protected software, used for research purposes only, then destroyed after completion of the transcription.

- Yes (1)
- No (2)

**How my information will be used after the project**

I understand that personal details, such as my name and email address, will not be revealed to anyone outside of the project.

- Yes (1)
- No (2)

I am happy for my anonymized, transcribed data to be stored and used by others for future research.

- Yes (1)
- No (2)

I understand and agree my data may be published as a journal article and may appear in other materials used to share the findings of the project.

- Yes (1)
- No (2)

I am happy for my e-mail address to be stored for up to 12 weeks so that I can receive a gift voucher upon study completion.

- Yes (1)
- No (2)

End of Block: Block 1

Start of Block: Block 2

Please confirm that you agree (consent) to take part in this research project by signing below.

- Name (1) __________________________________________________
- Email address (2) __________________________________________________
- Date of completion (3) __________________________________________________

End of Block: Block 2

**Supplementary Material 5: Topic guide for focus groups**

**Group 1: Members of the Public (Total: 60 minutes)**

1. **Introduction (5 minutes)**

- Welcome and thank participants for attending.
- Outline ground rules: open discussion, confidentiality, and respect for different opinions.
- Introductions: Name and where in the country they are calling from
- In this research project, we are doing a horizon scan to find what diagnostic tests for HPV that can be used outside of a clinical setting are ready to come on to the market.
- Horizon scanning is a method used by researchers to find out what types of health technologies (such as medicines and diagnostic tests) could potentially come into use within the health service in the future. This information can then be used to help inform policymakers in advance.
- Previously, you helped our research by filling in a survey; this helped us to know more about what features of the kits were most important (such as easy to understand instructions, or if the test was free)
- Now, we want to talk to you in more depth about what barriers there may be to people using a HPV test outside of a clinical setting
- We are not going to ask you for your personal experiences, but we recognise that your perspectives may be shaped by your past experiences
- The perspectives you give in this focus group will help us to put the findings of our wider research project into context, helping us to understand more about potential barriers to using HPV testing kits outside of a clinic
- Reminder that we will be talking very generally and not asking for personal experiences, though appreciate that perspectives may be shaped by this
- Let participants know that the session will be recorded, obtain their verbal consent for recording, and remind them that they can withdraw at any time

**BEGIN RECORDING**

1. **Perceived Acceptability (12 minutes)**

A really important thing for us to understand is how acceptable these tests are from the perspective of people who would potentially be using these.

- **Prompt:** Do you think the availability of an at-home test for HPV is a good thing?
- **Prompt:** What would encourage you to use an at-home test kit instead of visiting a clinic?
- **Prompt:** Do you have any concerns about people using the kit?
- **New Prompt:** How important are features like **free access**, **ease of use**, **clear instructions**, or **adequate follow-up** in your decision to use an at-home test?
  - What about **discretion** (being able to take the test privately and not having others know)?
  - What about **emotional concerns**, such as potential embarrassment?

1. **Barriers to Using HPV At-Home Test Kits (12 minutes) – Key Question 1**

It’s also important to understand if there are some barriers that would make it difficult for people, or some groups of people, to use these tests at home.

- - **Prompt:** What factors do you think might make it difficult for people to use an HPV test kit at home?
  - **Barriers to Probe:**
    - Personal characteristics, such as a person’s age or whether they have a disability (e.g. mobility issues)
    - A person’s culture
    - The languages someone speaks
    - Refer to [PROGRESS-Plus](https://methods.cochrane.org/equity/projects/evidence-equity/progress-plus) domains for further prompts if required

1. **Importance of Barriers (10 minutes) – Key Question 2**
   - Briefly remind people of the barriers they have discussed so far
   - **Prompt:** Which of these barriers are the most important and why?
   - **Prompt:** How likely are these barriers to prevent you from using the test? How could these barriers be addressed?
2. **Interaction with Healthcare Professionals (8 minutes)**
   - **Prompt:** What do you think healthcare providers need to know to understand and help with the challenges we've discussed?
   - **Prompt:** Is there any other support or information would you need to feel comfortable using the kit?
3. **Thought Experiment: Overcoming Material Barriers (8 minutes)**
   - **Scenario:** If practical barriers like cost and logistics were removed, would this change your view on using the test?
   - **Prompt:** What non-material barriers would still remain?
     - Reflect on non**-**material barriers previously mentioned but others include E.g.
     - Privacy
     - Stigma
     - PROGRESS-Plus factors such as culture, language, socioeconomic status
4. **Wrap-Up (5 minutes)**
   - Final thoughts or additional input.
   - Thank participants and explain how their perspectives will be used to shape the research that we are doing by helping to add further context to our work.
   - Remind participants that they will receive a £25 digital gift voucher for their time spent attending the focus group via email.

**Additional information for the chat if people want more information regarding HPV testing**

- **NHS:** [**https://www.nhs.uk/conditions/human-papilloma-virus-hpv/**](https://www.nhs.uk/conditions/human-papilloma-virus-hpv/)
- **Gov.uk:** [**https://www.gov.uk/government/publications/cervical-screening-support-for-people-who-find-it-hard-to-attend/cervical-screening-support-for-people-who-feel-anxious-about-attending**](https://www.gov.uk/government/publications/cervical-screening-support-for-people-who-find-it-hard-to-attend/cervical-screening-support-for-people-who-feel-anxious-about-attending)

**Group 2: Clinicians (Total: 60 minutes)**

1. **Introduction (5 minutes)**

- Welcome and thank participants for attending.
- Outline ground rules: open discussion, confidentiality, and respect for different opinions.
- Introductions: Name and where in the country they are calling from
- Briefly explain the purpose of the focus group: to gather opinions on the potential adoption of HPV at-home test kits and explore factors that might influence their use from a clinical perspective.
- Reminder that we will be talking very generally and not asking for personal experiences, though appreciate that perspectives may be shaped by this
- Let participants know that the session will be recorded, obtain their verbal consent for recording, and remind them that they can withdraw at any time

**BEGIN RECORDING**

1. **General Perception of HPV At-Home Test Kits (8 minutes)**

A really important thing for us to understand is how acceptable these tests are from the perspective of people who would potentially be recommending them to others.

- **Prompt:** Do you think the availability of an at-home test for HPV is a good thing?
- **Prompt:** What would encourage you to tell your patients about at-home test kits as an alternative to visiting a clinic?
- **Prompt:** Do you have any concerns about people using the kit?
- **New Prompt:** How important would features like **free access**, **ease of use**, **clear instructions**, or **adequate follow-up** in your decision to tell your patients about at-home tests?
  - What about **discretion** (being able to take the test privately and not having others know)?
  - What about **emotional concerns**, such as potential embarrassment?

1. **Clinical Efficacy and Reliability (10 minutes)**
   - **Prompt:** Would you have any concerns about the potential accuracy of HPV at-home test kits compared to in-clinic tests?
   - **Prompt:** How comfortable would you feel recommending these kits, especially for patients less likely to attend clinics?
2. **Barriers to Implementation (12 minutes) – Key Question 1**

It’s also important to understand if there are some barriers that would make it difficult for people, or some groups of people, to use these tests at home.

- - **Prompt:** What barriers do you think would prevent patients from engaging with at-home HPV testing?
  - **Barriers to probe if no initial responses:**
    - Cultural factors
    - Personal characteristics such as age or disability
    - Someone’s educational background
    - What languages someone speaks
    - Socioeconomic status
    - Other PROGRESS-Plus factors if applicable

1. **Importance of Barriers (8 minutes) – Key Question 2**
   - **Prompt:** Which of these barriers do you think are the most important?
   - **Prompt:** How likely are these barriers to prevent people from using at-home test kits?
   - **Prompt:** How important is it to understand these sorts of challenges in order for you to consider recommending using a one of these tests?
2. **Thought Experiment: Overcoming Material Barriers (7 minutes)**
   - **Scenario:** If practical barriers like cost and logistics were removed, would this change your view on recommending the HPV at-home test?
   - **Prompt:** What non-material barriers would still remain?
     - Reflect on non**-**material barriers previously mentioned but others include E.g.
     - Privacy
     - Stigma
     - [PROGRESS-Plus](https://methods.cochrane.org/equity/projects/evidence-equity/progress-plus) factors such as culture, language, socioeconomic status

**Clinician Role in Facilitating Adoption (5 minutes)**

- **Introductory context:** Clinicians play a key role in patient education and adoption of new testing methods.
- **Prompt:** What role do you see yourself playing in helping patients feel comfortable with using HPV at-home test kits?
  - What educational resources or support systems would be helpful for clinicians in this regard?
- **Prompt:** How can the healthcare system better support clinicians in recommending these tests?
  - Would ongoing training, easy access to reliable resources, or a streamlined referral system help?

1. **Wrap-Up (5 minutes)**
   - Final thoughts or additional input.
   - Thank participants and explain how their perspectives will be used to shape the research that we are doing by helping to add further context to our work.

**Supplementary Material 6: Discussion of main findings of survey stratified by respondent role**

**Design principles**

Across respondent groups, the features most consistently prioritised were free access to tests and accuracy comparable to clinician-administered screening. For clinicians, these were the top-ranked features (43%, n=15 for each), followed by ease of use (37%, n=13) and free shipping for analysis (31%, n=11). Members of the public similarly emphasised free access (~47%, n=22) and accuracy (~45%, n=21), with adequate follow-up care (~38%, n=18) and ease of access (30%, n=14) also highlighted. Researchers placed greatest importance on accuracy (~71%, n=10) and accessibility (~50%, n=7), with fewer prioritising free access (37%, n=5). Other features—such as follow-up care, discretion, data security, and reduction of pain or discomfort—were mentioned less frequently across groups.

**PROGRESS-Plus domains**

Nearly 43% (n=15) of clinicians highlighted the importance of cultural factors on people’s ability or willingness to take a self-sampled test for HPV, as did ~43% (n=6) of researchers. Members of the public also suggested that cultural factors were an important consideration (~43%, n=20), but considered personal characteristics such as age and disability to have the greatest potential impact (~49%, n=23). Forty percent (n=14) of the clinicians who responded suggested these other characteristics were significant, as did ~50%, (n=7) of the researchers who responded The languages a person spoke was considered a potentially important consideration by both clinicians (~34%; n=12) and members of the public (~37%; n=13) and researchers (~36%; n=5). Level of education (~37%, n=13), language (~34%, n=12), and socioeconomic status (~28%, n=10) were also recognised as important factors by clinicians.

**Supplementary Material 7: Additional responses from free-text section of survey**

Twenty respondents provided additional perspectives as free text within the survey. These responses came from eight clinicians, seven members of the public, four from people who identified in another role and one researcher.

One clinician expressed concerns about the tests’ use beyond their current role as healthcare professionals in leading cervical screening, suggesting they could cause unnecessary anxiety, distress and confusion. They questioned how healthcare professionals would appropriately time and frame conversations with patients receiving positive test results, particularly regarding infection risk and the potential for malignancy Another clinician highlighted that most unvaccinated individuals are likely to contract HPV at some stage, considering it a normal occurrence. They questioned the value of testing asymptomatic individuals, noting that visible HPV lesions already indicate infection. Diagnosing asymptomatic individuals, they argued, could lead to unnecessary stress and stigma, which should be avoided. Concerns were also raised that moving tests outside of clinical settings could reduce opportunities to address important issues—such as sexual and domestic violence—that are often identified during in-clinic visits. Clinicians also raised questions about how follow-up care would be organised and noted that there is currently no treatment for HPV available through the NHS, which should be clearly communicated to patients.

Members of the public also shared additional perspectives at the end of the survey (n=7), suggesting that instructions for self-sampled HPV tests should be in multiple languages and use discreet packaging. One respondent also noted that some women who are not sexually active may find using some tests, such as vaginal swabs, less acceptable.

**Supplementary Material 8: Minor themes discussed within the focus groups**

**Design principles**

*Privacy and autonomy*

Members of the public expressed a preference for keeping the results of the test between themselves and their GP. As two members of the public noted:

*“[…] If you could tick a box when you send the test or if you could say do not contact me. I will contact you in 3 weeks, or whatever how long it takes […]”*

*“I think you've got to be very, very careful. Sending these results through the post, had somebody die of cervical cancer or whatever. So, you've got to be very careful how you're going to give the results out”*

*Clarity and ease of use*

Finally, both clinicians and members of the public agreed on the need for clear instructions and patient information. Clinicians suggested that a negative HPV result using a test outside of the clinic may not preclude the need to seek medical advice if symptoms arise. They also suggested this clarity would help prevent misunderstanding and ensure that symptoms potentially unrelated to HPV are appropriately managed. Regarding the clarity of instructions, members of the public specifically suggested that integrating technology, such as QR codes leading to instructions in multiple languages and instructional videos, could enhance understanding and accessibility for first-time users of self-sampled tests. Two members of the public highlighted Self-sampled diagnostics for other conditions (such as COVID-19 and colorectal cancer) as examples of clear test instructions.

*Accuracy and trustworthiness*

While members of the public briefly discussed the importance of the diagnostic accuracy of self-sampled HPV tests, clinicians placed greater emphasis on reliability. The clinicians noted that clinic-based swab tests are generally accurate and straightforward for most people, with one highlighting the ideal of a reliable self-sampled test that could diagnose high-risk types of HPV. Another stressed the need for comparative data on the accuracy of self-sampled HPV tests versus clinic-based smear tests, expressing concerns that implementing an ineffective test could erode trust in the screening process. However, another clinician more favourably compared the potential for self-sampled tests for HPV with self-sampled diagnostic tests for other STIs.

“*I think. I mean, they're pretty good at taking their gonorrhoea and Chlamydia swabs. They're pretty accurate. And yeah, not many people have issues taking the swab. So, I think from an accuracy point of view. I wouldn't have any particular concerns.”* – Clinician

**Equality considerations**

*Community engagement and time-dependent factors*

Members of the public also noted other relational factors that may discourage people from using self-sampled tests, including potential value judgements (such as “promiscuity”) and the potential impacts on long-term relationships if the test was positive, given that HPV is an STI.

Members of the public suggested that HPV self-sampling initiatives should consider social dynamics and involve community organisations and leaders to promote awareness. They emphasised the importance of incorporating young people’s perspectives in outreach, as younger individuals may perceive health risks as less immediate. One member of the public suggested that a way to raise awareness was through mobile clinics, where professionals could assist with test kit instructions, while another proposed involving community organisations and leaders with connections to groups less likely to participate in screening.

*“I think, being able to use like community organisations that are like more connected with different groups who might be excluded like more minority groups that might be good, because they have better access to them, and they might be able to better facilitate reaching those groups and engaging with them to take up the tests and explaining to them like the benefits and everything”*

The members of the public described how these organisations could effectively promote and advocate for the benefits of testing, reflecting the public’s preference for collaborative approaches to support diverse groups in HPV screening.

**Other concepts**

Participants in both focus groups discussed concepts related to self-sampled HPV tests unrelated to design or accessibility. While clinicians suggested that the increased accessibility offered by self-sampling could potentially ease strain on GP resources and improve cost-effectiveness, one member of the public suggested that GP surgeries may be reluctant to manage self-sampled tests due to logistical requirements, such as storage and inventory management. Additionally, some clinicians also highlighted the importance of HPV screening other parts of the body (e.g. oral screening for mouth cancer); this was echoes by two members of the public, who mentioned HPV screening for throat cancer. As one clinician noted:

*“[…] I think the oral screening. I think that's really difficult, because whilst there's a very definite association with oral cancers and certain types of HPV. Most people who have oral HPV are not going to get an oral cancer. But we also know that you get genital skin cancers that are HPV-related. And so, you can imagine that people will want to know. Or that, you know, they want to understand why, why it doesn't apply, for example, to testing other parts of the genital area or the mouth.”*

There were some discussions surrounding the potential impact on the frequency of testing for HPV. Some clinicians discussed the potential for over-testing for HPV should self-sampled test kits become available but, by contrast, one member of the public suggested self-sampled tests for HPV could be an option for those that choose to screen more regularly.
